# Desafios e Aplicações dos Testes Genéticos na Cardiomiopatia Dilatada: Genótipo, Fenótipo e Implicações Clínicas

**DOI:** 10.36660/abc.20230174

**Published:** 2023-11-14

**Authors:** Silas Ramos Furquim, Bianca Linnenkamp, Natália Quintella Sangiorgi Olivetti, Fernando Rabioglio Giugni, Layara Fernanda Vicente Pereira Lipari, Fernanda Almeida Andrade, José Eduardo Krieger

**Affiliations:** 1 Hospital das Clínicas Faculdade de Medicina Universidade de São Paulo São Paulo SP Brasil Instituto do Coração do Hospital das Clínicas da Faculdade de Medicina da Universidade de São Paulo, São Paulo, SP – Brasil

**Keywords:** Cardiomiopatia Dilatada, Genética, Testes Genéticos

## Abstract

Os testes genéticos para cardiomiopatia dilatada (CMD) apresentam uma positividade de até 40%, mas há uma grande heterogeneidade genética e outros desafios decorrentes de expressividade variável e penetrância incompleta. O heredograma é fundamental para diferenciar os casos de CMD esporádica e familiar, por meio da avaliação do histórico familiar. A CMD familiar apresenta um rendimento maior nos testes genéticos, mas a CMD esporádica não exclui a possibilidade de causa genética. Alguns genes têm fenótipos específicos, sendo o gene da Lamina ( *LMNA* ) o mais fortemente associado a um fenótipo de arritmias malignas e quadros de insuficiência cardíaca (IC) avançada. A presença de uma variante genética causal também pode ajudar na avaliação prognóstica, identificando quadros mais graves e com menores taxas de remodelamento reverso em comparação com indivíduos com genótipo negativo. As diretrizes atuais recomendam a avaliação e aconselhamento genético em indivíduos com CMD, além do rastreamento em cascata nos familiares de primeiro grau nos casos em que há uma ou mais variantes identificadas, sendo uma oportunidade para o diagnóstico e tratamento precoces. Familiares com genótipo positivo e fenótipo negativo são candidatos à avaliação seriada, com periodicidade que varia conforme a idade. O genótipo também auxilia na indicação individualizada de cardiodesfibrilador implantável e em recomendações quanto à atividade física e planejamento familiar. Estudos em curso esclarecem progressivamente os detalhes das relações genótipo/fenótipo de um grande número de variantes e fazem com que a genética molecular esteja cada vez mais presente na prática clínica.

## Introdução

A cardiomiopatia dilatada (CMD) é uma condição em que há dilatação do ventrículo esquerdo (VE) e redução da fração de ejeção (FE), na ausência de causas secundárias, como isquemia miocárdica, hipertensão arterial, valvopatias primárias ou cardiopatias congênitas.^1 - 4^ A CMD é relativamente comum, com prevalência que varia de 1/250 a 1/500 na população geral,^4 , 5^ sendo a principal causa de transplante cardíaco em todo o mundo.^6^ Quando a etiologia da CMD não é bem definida, a investigação de uma causa genética se faz relevante. Em até 40% dos pacientes com CMD é encontrada uma variante genética patogênica ou provavelmente patogênica que poderia explicar o fenótipo cardíaco, o que ressalta a importância da realização do teste genético nessa população. No entanto, a interpretação do resultado do teste genético na CMD pode ser desafiadora, considerando a ampla variedade de genes envolvidos, a incompleta penetrância genética no fenótipo clínico e a grande heterogeneidade de expressão das variantes na manifestação clínica.^3 , 5^ A maioria dos genes associados à CMD não causa exclusivamente esse fenótipo, estando relacionados com outras cardiopatias, como cardiomiopatia hipertrófica (CMH), cardiomiopatia arritmogênica ou canalopatias, podendo causar sobreposição de fenótipos.^5 , 7 , 8^ Identificar uma variante patogênica relacionada à CMD é fundamental para reforçar o diagnóstico, refinar a avaliação prognóstica e, nos casos de teste positivo em pacientes assintomáticos, abrir a possibilidade do rastreamento clínico, diagnóstico e tratamento precoces. A indicação do melhor método de teste genético a ser solicitado, a correta interpretação das variantes encontradas e as implicações práticas dos resultados são desafios atuais da Medicina de Precisão em Cardiologia. Essa revisão abordará esses aspectos, à luz de publicações recentes na área.

### Definição de CMD esporádica e familiar

**Figura 1:**
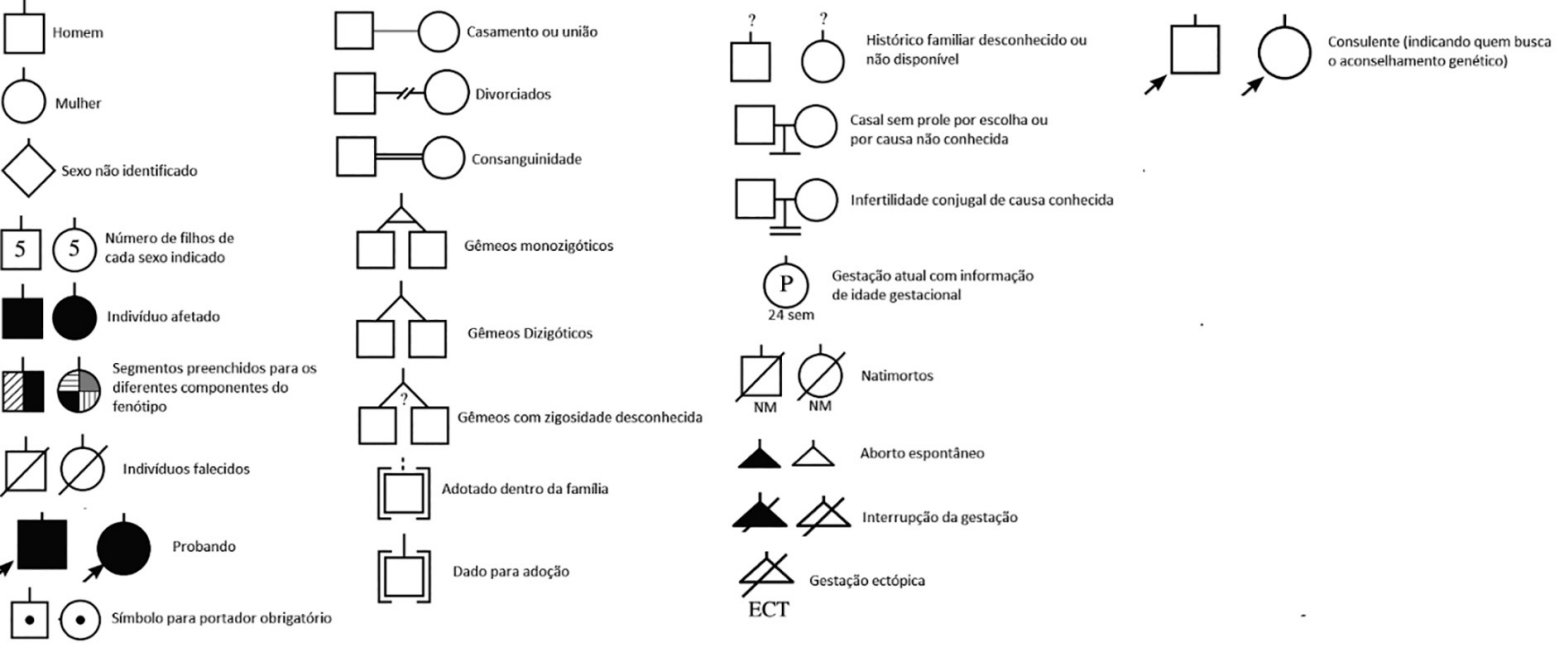
– Símbolos universais utilizados na construção do heredograma.Fonte: Kim et al. ^43^

A CMD familiar é definida quando dois ou mais familiares apresentam critérios para CMD ou o caso índice com CMD tem um familiar de primeiro grau que apresentou morte súbita em idade menor que 35 anos ou confirmação por autópsia de CMD.^4 , 9 , 10^ Quando a CMD é familiar, a probabilidade de encontrar uma variante patogênica associada ao fenótipo é maior e, portanto, o rendimento do teste genético é mais alto. No entanto, mesmo que a CMD seja esporádica, não se exclui causa genética, considerando-se a possibilidade de uma mutação *de novo.* ^8 , 9^ A diretriz da *American Heart Association* (AHA) / *American College of Cardiology* (ACC) de 2022 recomenda que seja feita a avaliação de história familiar de pelo menos três gerações, idealmente, em forma de heredograma.^3^ Na tabela 1 e na figura 1 encontram-se orientações básicas para a construção de um heredograma.

**Tabela 1:** – Principais informações e regras necessárias para construção do heredograma


Identificar com nome do probando (paciente em atendimento), data de nascimento, idade do probando durante avaliação e data do atendimento.
Construir o heredograma com informações de pelo menos três gerações a partir do probando e incluir familiares de primeiro grau do probando (pais, irmãos e prole); familiares de segundo grau (avós, tios e tias, sobrinhos e sobrinhas e netos).
Indicar local de origem e ascendência da família do probando, além de questionar se há consanguinidade na família. Nem sempre a consanguinidade é conhecida, no entanto, deve-se suspeitar desta possibilidade quando os genitores forem de município com poucos habitantes.
Identificar doenças conhecidas e outros achados através de legendas.
Posicionar indivíduos; mais velhos devem ficar mais à esquerda e os mais novos à direita.
Indicar a idade e a causa *mortis* de indivíduos falecidos.
Numerar as gerações através de numerais romanos a fim de facilitar a identificação de indivíduos na família.
Sinalizar indivíduos que tenham realizado teste genético e indicar o resultado.

### Quando solicitar teste genético

A recomendação de testagem genética se encontra nas últimas diretrizes de insuficiência cardíaca da AHA/ACC, da *European Society of Cardiology* (ESC) e da Sociedade Brasileira de Cardiologia (SBC), indicando a avaliação em pacientes com CMD, acompanhada do aconselhamento genético.^3 , 4 , 11^ Pode ser útil em casos limítrofes, como para distinguir CMD de cardiomiopatia periparto ou cardiomiopatia arritmogênica de VE, por exemplo. A testagem genética também pode encontrar diagnósticos diferenciais e que apresentam tratamento específico, como a amiloidose cardíaca que, em fases mais tardias, pode se apresentar com redução da FE.^12^ Também está indicado para decidir intervenções, como implante de cardiodesfibrilador (CDI) como profilaxia primária.^13^ Importante salientar que a testagem genética deve ser realizada no membro da família mais afetado, com fenótipo bem definido, para aumentar o rendimento do teste e, posteriormente, desencadear o rastreamento familiar em cascata.^14^

### Qual teste genético solicitar

Há uma diversidade de genes relacionadas à CMD, e, para uma avaliação eficiente, é necessário um método que analise os principais genes envolvidos de forma simultânea e rápida. O surgimento de uma nova tecnologia de sequenciamento, denominada *NGS (next generation sequencing)* permitiu o sequenciamento paralelo em massa de diversos genes, o que tornou estes testes mais acessíveis e sua inclusão na rotina do especialista.

A utilização de painéis de NGS para análise de genes pré-selecionados relacionados a determinado fenótipo revolucionou a prática clínica por conciliar maior agilidade e eficiência e é o método recomendado pelo consenso de especialistas de teste genético em cardiologia.^15^ A lista de genes deve ser atualizada com base no conhecimento científico e é imprescindível que o painel de NGS escolhido para investigação de CMD contenha pelo menos os genes mais frequentemente associados a esta condição, representados na tabela 2 .^15 , 16^

**Tabela 2:** – principais variantes que devem ser pesquisadas na cardiomiopatia dilatada

Gene	Frequência	Gene	Frequência	Gene	Frequência
*TTN*	18-25%	*MYBPC3*	2%	*DES*	<1%
*DSG2*	4–15%	*FLNC*	0–3%	*TMEM43*	<1%
*DSP*	1-13%	*ACTC1 *	<1%	*TAZ*	Desconhecida
*PLN*	0–12%	*LDB3*	<1%	*BAG3*	Desconhecida
*LMNA*	6%	*TNNC1*	<1%	*RBM20*	Desconhecida
*MYH6*	4%	*TNNI3*	<1%	*DSC2*	Desconhecida
*MYH7*	4%	*TNNT2*	<1%	*DMD*	Desconhecida
*SCN5A*	0–2%	*TPM1*	<1%	*EMD*	Desconhecida

Exames mais amplos, como o sequenciamento de exoma e o sequenciamento de genoma, também se baseiam na tecnologia NGS, e analisam todos os genes conhecidos; no entanto apresentam como desvantagens custo e tempo de processamento elevados.^5 , 16^

### Rendimento do teste genético na CMD

**Figura 2:**
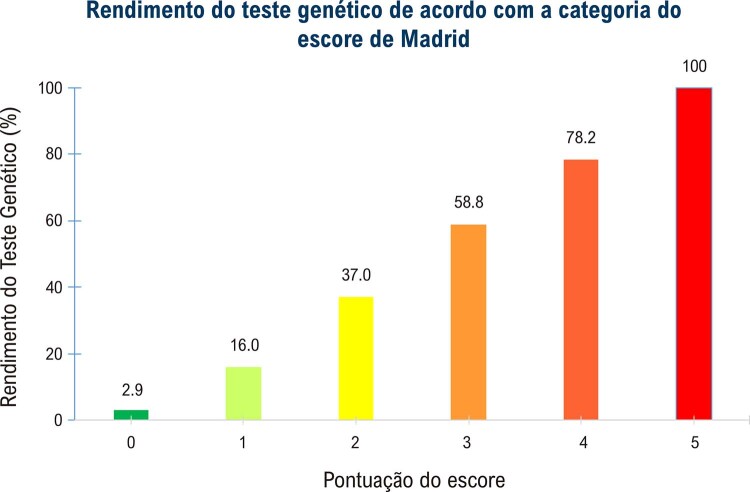
–  Rendimento do teste genético de acordo com a categoria do escore de Madrid. Fonte: Adaptado de Escobar-Lopez. ^17^

O rendimento da testagem genética para identificação de uma variante patogênica ou provavelmente patogênica relacionada à CMD varia de 15% a 40%,^3^ dependendo de fatores como história familiar positiva, presença de comorbidades e características do eletrocardiograma (ECG).^17^ Escobar-Lopez et al. propuseram um escore para estimar a probabilidade de positividade da testagem genética, denominado Escore de Madrid ( Tabela 3 ). Este escore avalia a presença de miopatia esquelética, história familiar de CMD, baixa voltagem no ECG, ausência de hipertensão e ausência de bloqueio de ramo esquerdo no ECG. A presença de 4 ou mais desses fatores pode levar a uma taxa de positividade do teste genético de 79% ( Figura 2 ).^17^

**Tabela 3:** – Escore de Madrid. Preditores de positividade do teste genético na CMD

Preditores de Teste Genético Positivo	
Miopatia esquelética	1 ponto
História familiar de CMD	1 ponto
Baixa voltagem no ECG	1 ponto
Ausência de hipertensão e ausência de BRE	1 ponto
Ausência de BRE	1 ponto
**Pontuação do escore: 0 a 5 pontos**	

CMD: cardiomiopatia dilatada; ECG: eletrocardiograma; BRE: bloqueio de ramo esquerdo. Fonte: Adaptado de Escobar-Lopez L. ^17^

### A interpretação das variantes encontradas

É importante ressaltar que a presença de uma variante genética deve ser avaliada com muita cautela. Encontrar uma variante genética em gene de cardiomiopatia, por si só, não é suficiente para afirmar causalidade em relação ao fenótipo clínico. Ao realizar um teste genético, mesmo em população saudável, a presença de variantes genéticas em genes relacionados a cardiomiopatia é comum. Deve-se levar em consideração que as variantes podem ser benignas. Portanto, um ponto crucial é a determinação da patogenicidade da variante. Para definir a patogenicidade de uma variante, é necessário avaliar a força da associação entre o gene e a cardiopatia, podendo-se utilizar como apoio uma ferramenta que faz a curadoria desta relação, o ClinGen (www.clingenome.org) e seguir os critérios propostos pelo *American College of Medical Genetics and Genomics* (ACMG).^18^ A determinação da patogenicidade das variantes é complexa e inclui várias informações, como a frequência da variante em bancos de dados genéticos populacionais, como o Genome Aggregation Database (GnomAD) e o **A** rquivo **Bra** sileiro **O** nline de **M** utações (ABraOM), as características bioquímicas, predições em sílico, investigação de relatos prévios relacionando a variante à doença, dados alélicos e dados de segregação familiar. É importante considerar o tipo de mutação ( *missense* , inserção, deleção, *nonsense* ) e o efeito da variante na proteína (encurtamento, parada precoce, alteração em sua fosforilação). Deve-se considerar, também, se o mecanismo de patogenicidade descrito para aquele gene é compatível com o efeito da variante na proteína.^18^

As variantes podem ser classificadas em cinco categorias: classe 5 (patogênica), classe 4 (provavelmente patogênica), classe 3 (variante de significado incerto), classe 2 (provavelmente benigna) ou classe 1 (benigna).^18^ As variantes patogênicas são consideradas causais, enquanto as provavelmente patogênicas possuem uma chance de 90% de serem causais. Já as variantes de significado incerto, comumente chamadas de VUS, do inglês *variant of uncertain significance,* representam uma área de maior incerteza quanto à patogenicidade que pode variar de 10 a 90% de serem causais ( Tabela 4 ).^18^ Algumas estratégias para reclassificar uma VUS incluem a realização de estudos funcionais que testam o efeito da variante na proteína, a segregação familiar da variante em parentes de primeiro grau e a pesquisa periódica na literatura científica.^18^ É importante ressaltar que a classificação de variantes é um processo dinâmico que depende do conhecimento atualizado sobre a variante em questão. Por isso, reclassificações são constantes e a busca por novas informações é essencial.

**Tabela 4:** – Classificação das variantes segundo ACMG

Classificação da patogenicidade	classe	probabilidade
Benigna	classe 1	< 5%
Provavelmente Benigna	classe 2	< 10%
Variante de significado incerto	classe 3	10 - 90%
Provavelmente patogênica	classe 4	> 90%
Patogênica	classe 5	> 95%

ACMG: American College of Medical Genetics and Genomics. Fonte: Richards et al. ^18^

### Diferentes genes implicados na CMD

Ao contrário da CMH, em que 70% das variantes estão presentes nos genes *MYH7* e *MYBPC3,* ^19^ a CMD apresenta uma maior heterogeneidade, com mais de 50 genes descritos em associação ao fenótipo.^7^ Esses genes codificam proteínas que atuam em diferentes estruturas do cardiomiócito ( Tabela 5 ). Por exemplo, a Lamina A/C ( *LMNA* ) e a RNA-binding motif protein-20 ( *RBM20* ) são encontradas no núcleo celular e estão presentes em 8% dos pacientes com CMD.^10 , 20^ No retículo sarcoplasmático, o fosfolambam ( *PLN* ) é encontrado e, quando não fosforilado, inibe o retículo sarcoplasmático Ca2+-ATPase ( *SERCA* ).^10 , 21^ No citoesqueleto, as variantes nos genes da Filamina C ( *FLNC* ), desmina ( *DES* ) e distrofina ( *DMD* ) correspondem a aproximadamente 11% dos casos.^20^ Já no sarcômero, encontram-se genes como *TTN, MYH7, TNNT2, TPM1* e *MYBPC3* .

**Tabela 5:** – Descrição de genes envolvidos na CMD, proteínas codificadas e região do cardiomiócito onde se encontram

Gene	Proteína	Região afetada
*LMNA*	Lamina A/C	Envelope nuclear
*RBM20*	RNA-binding motif protein-20	Núcleo
*PLN*	Fosfolambam	Retículo sarcoplasmático
*FLNC*	Filamina C	Citoesqueleto
*DES*	Desmina	Citoesqueleto
*DMD*	Distrofina	Citoesqueleto
*TTN*	Titina	Sarcômero
*MYH7*	Cadeia pesada da beta miosina	Sarcômero
*TNNT2*	Troponina T	Sarcômero
*TPM1*	Tropomiosina	Sarcômero
*MYBPC3*	Proteína C 3 ligadora da miosina	Sarcômero
*SCN5A*	Canal de sódio voltagem dependente subunidade alfa 5	Membrana celular

O gene *TTN,* que codifica a proteína titina, é o mais comumente implicado na CMD, sendo que variantes truncadas em *TTN* podem corresponder a 25% das CMD familiares e 18% das esporádicas.^22^ As variantes dos genes *MYH7, TNNT2* e *TPM1* têm uma prevalência de 5 a 10%,^23^ e *MYBPC3* , embora seja mais específico para CMH, também é encontrado na CMD.^24^ Além disso, na membrana celular se encontra o principal canal de sódio do coração, codificado pelo gene *SCN5A* . Embora as variantes desse gene sejam bem descritas em arritmias, como as síndromes do QT longo tipo 3 e a Síndrome de Brugada, variantes do tipo “ *missense”* também são descritas em associação ao fenótipo de CMD.^10^ A ampla gama de genes implicados, juntamente com a grande sobreposição fenotípica, torna desafiadora a avaliação genética da CMD.^20^

### Manifestações clínicas em variantes específicas

As manifestações clínicas de variantes específicas da CMD são bastante heterogêneas, o que significa que um mesmo gene pode ser responsável por diferentes fenótipos. Por exemplo, variantes no gene *TNNT2* podem se manifestar como CMH, dilatada ou restritiva. Essa variação clínica pode ser explicada pela epigenética que são as interações entre a variante específica com a genética, de cada indivíduo e fatores externos, como hipertensão, alcoolismo, estilo de vida, exercício físico, taquicardia, quimioterapia ou inflamação.^10 , 20^

Alguns genes, como *PLN, FLNC* e *LMNA* , estão mais diretamente relacionados a fenótipos agressivos, com maior potencial de arritmias ventriculares, morte súbita e pior prognóstico. O gene *LMNA* é particularmente associado a um fenótipo específico, que atualmente tem sido chamado de laminopatia, caracterizado por disfunção ventricular esquerda precoce (30 – 40 anos), distúrbios de condução elétrica em idade precoce (como bloqueio atrioventricular total), fibrilação atrial em jovens, arritmia ventricular complexa e alto risco de morte súbita cardíaca, mesmo na ausência de disfunção ventricular esquerda.^9 , 20 , 25 , 26^ Como resultado dessa associação, existem recomendações específicas para restrição à atividade esportiva competitiva e indicações específicas de profilaxia primária com CDI nesta população.^13^

Além disso, variantes patogênicas e provavelmente patogênicas em genes desmossomais, como da desmoplaquina ( *DSP* ), foram inicialmente relacionadas à cardiomiopatia arritmogênica de ventrículo direito, mas novas evidências também demonstram associação com fenótipo de CMD.^27^ Junto com os genes *FLNC* e *LMNA* , são as principais causas de cardiomiopatia arritmogênica na forma predominante de VE, caracterizada por arritmias ameaçadoras à vida que ocorrem de forma mais precoce, desproporcional ao grau de disfunção ventricular esquerda.^20^

O gene *TTN* codifica a proteína titina, que é importante para a elasticidade passiva do tecido miocárdico. Variantes de perda de função neste gene desempenham um papel bem estabelecido na patogênese da CMD, enquanto variantes do tipo *missense* são frequentes e, na maioria das vezes, consideradas benignas.^20^

Finalmente, o gene *DMD* , relacionado às distrofias musculares, está associado a uma manifestação clínica típica de fraqueza muscular progressiva. A incidência de cardiomiopatia aumenta com a idade, especialmente em homens, chegando a mais de 90% aos 18 anos. O ECG apresenta um padrão clássico com ondas R altas e aumento da amplitude R/S em V1, ondas Q nas derivações precordiais esquerdas, eixo desviado para direita ou bloqueio completo do ramo direito.^28^

### Impacto prognóstico do teste genético

Pacientes com CMD e variantes patogênicas ou provavelmente patogênicas apresentam piores desfechos clínicos, especialmente quanto ao risco de arritmias malignas e IC avançada, em comparação com pacientes com CMD e genótipo negativo. O risco é maior principalmente naqueles com FE ≤ 35%.^29^ No entanto, há variações entre os genes afetados; *PLN, LMNA* e *FLNC* apresentam maior risco de arritmias malignas, mesmo com FE > 35%.^29^ Pacientes com laminopatias têm mortalidade em torno de 12% em 4 anos e maior necessidade de transplante cardíaco aos 45 anos.^9 , 20 , 25^ As variantes em *FLNC* tem associação com uma alta predisposição a arritmias ventriculares malignas e morte súbita, com taxas de 15-20% de arritmias ventriculares ou morte súbita em 5 anos de seguimento e 6% de mortalidade.^30^ As variantes em *PLN* estão associadas a formas mais graves de cardiomiopatia, com arritmias malignas, rápida progressão para IC avançada e necessidade de transplante cardíaco.^20^ Entre os genes mencionados, variantes em *TTN* apresentam melhor prognóstico, com menores taxas de arritmias malignas e maior incidência de remodelamento reverso (RR).^29^

### Remodelamento reverso

A CMD é uma doença dinâmica, que pode apresentar melhora na FE em resposta ao tratamento, processo conhecido como RR. Essa melhora ocorre em cerca de 40% dos casos.^31^ Estudos têm investigado a relação entre as bases genéticas da CMD e o RR, e demonstram que pacientes com genótipo positivo apresentam taxas de RR inferiores, especialmente aqueles portadores de variantes desmossomais ( *PKP2, DSG2, DSC2, JUP, DSP* ), variantes relacionadas ao envelope nuclear (LMNA) e variantes em genes sarcoméricos ( *MYH7, MYBPC3* ).^29^

Diante disso, o conhecimento da genética dos pacientes pode trazer informações importantes para a estratificação de risco e predição prognóstica na CMD, desencadeando um acompanhamento mais frequente, otimização da terapêutica e avaliação mais precoce para indicação de dispositivos de assistência ventricular mecânica ou transplante cardíaco, se ocorrer evolução clínica desfavorável da IC.

### Rastreio familiar e acompanhamento

Uma vez identificada uma variante patogênica ou provavelmente patogênica no caso índice, deve-se seguir o rastreamento em cascata dos familiares, conforme também recomendado pelas principais diretrizes de insuficiência cardíaca.^3 , 4 , 11^ Tal avaliação tem como objetivo identificar familiares sob o risco de desenvolvimento da condição por serem portadores da variante e permite detectar indivíduos com doença instalada em fase precoce assintomática. Em um estudo que identificou variantes patogênicas ou provavelmente patogênicas em pacientes transplantados cardíacos por CMD, o rastreamento familiar identificou variantes patogênicas em 39,6% dos familiares, e destes, a maioria (52,6%) não tinha fenótipo clínico da doença.^32^ O diagnóstico em familiares é útil para iniciar o tratamento precoce, evitando a progressão da doença e morte súbita.^3 , 4 , 14^ Os principais dados estão resumidos na figura central .

**Figura Central:**
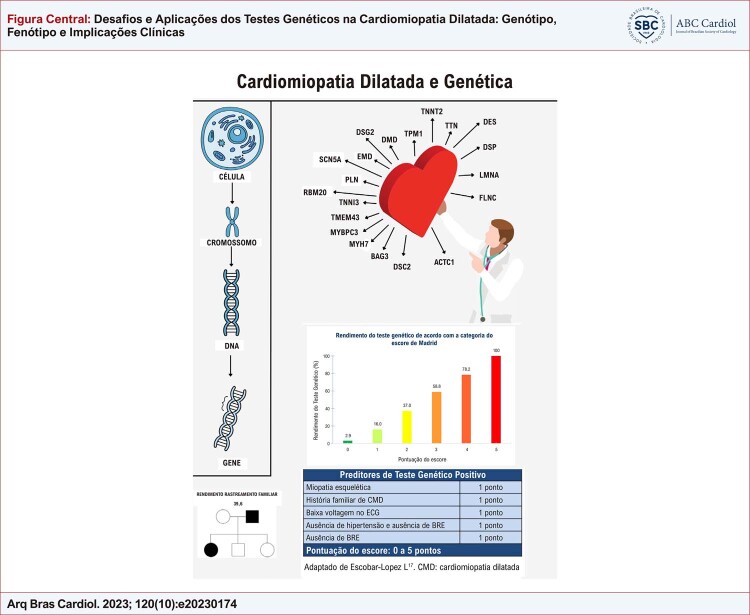
: Desafios e Aplicações dos Testes Genéticos na Cardiomiopatia Dilatada: Genótipo, Fenótipo e Implicações Clínicas

ACTC1:actin alpha cardiac muscle 1; BAG3: BAG cochaperone 3; DNA: Deoxyribonucleic Acid; DES: Desmin; DMD: Distrofin; DSC2: desmocollin 2; DSG2: desmoglein 2; DSP: desmoplakin; EMD: emerin; FLNC: Filamin C; LMNA: Lamin A/C; MYBPC3:myosin binding protein C3; MYH7: myosin heavy chain 7; PLN: phospholamban; RBM20: RNA-binding motif protein-20; SCN5A: sodium voltage-gated channel; alpha subunit 5; TMEM43:transmembrane protein 43; TNNI3:troponin I3; TNNT2: Troponin T; TPM1: Tropomiosin; TTN: Titin.

O teste genético é recomendado em familiares de primeiro grau a partir dos 10-12 anos de idade quando uma variante específica é identificada no caso índice.^20 , 33^ No entanto, esta idade pode ser individualizada e a testagem antecipada de acordo com a idade de diagnóstico de outros familiares afetados. Nesse caso, a técnica de sequenciamento por Sanger pode ser usada, ao invés do NGS, pois pesquisa apenas a variante de interesse, economizando tempo e recursos.^20^

A idade é um fator importante para o desenvolvimento das cardiomiopatias,^14^ em uma coorte avaliada com variantes truncadas em *TTN* , por exemplo, a penetrância aos 40 anos de idade foi acima de 95%.^22^ Portanto, a identificação de um familiar com genótipo positivo e fenótipo negativo requer avaliação seriada principalmente enquanto estiver na idade de maior risco de desenvolver as alterações fenotípicas.^3 , 14 , 20^

Além do teste genético, os familiares também necessitam de uma consulta clínica, com aconselhamento genético, anamnese e exame físico, associados a exames complementares, como ecocardiograma, eletrocardiograma^10^ e, se necessário, holter e teste de esforço. O resultado do teste genético pode, inclusive, determinar a frequência da avaliação clínica no seguimento e necessidade de repetição desses testes.^4^ A periodicidade da avaliação de familiares com genótipo positivo e fenótipo negativo varia conforme a idade e a cardiopatia de interesse. A *Heart Failure Society of America* sugere uma periodicidade para diferentes faixas etárias conforme a tabela 6 .^14^

**Tabela 6:** – Periodicidade da avaliação clínica em familiar de primeiro grau de caso índice, com variante genética relacionada à CMD identificada

Faixa etária	0-5 anos	6-12 anos	13-19 anos	20-50 anos	>50 anos
Periodicidade da avaliação	Anualmente	1-2 anos	1-3 anos	2-3 anos	5 anos

Não existem recomendações para iniciar o tratamento medicamentoso em pacientes portadores de variantes genéticas e fenótipo negativo; uma exceção é a presença de variante na *LMNA* que indica a colocação de CDI mesmo em fases assintomáticas, na presença de outros critérios abordados mais adiante.

Por outro lado, familiares com genótipo negativo tem baixa probabilidade de desenvolvimento da doença e não tem indicação de avaliação seriada, minimizando o peso de um possível acometimento cardíaco futuro.^14^

Quando não se encontra variante patogênica ou provavelmente patogênica no caso índice, os familiares de primeiro grau não devem realizar o teste genético e tem indicação de avaliação clínica seriada enquanto estiverem em idade de risco de desenvolver o fenótipo.^33^

Permanecem, no entanto, situações desafiadoras, como penetrância incompleta, expressão de fenótipos diferentes entre membros da mesma família e a incerteza da fase da vida em que a doença se manifestará.

### Terapias direcionadas e recomendações específicas

A presença de variantes genéticas específicas pode acarretar riscos particulares, havendo, assim, recomendações distintas de tratamento e manejo. Esse é o caso das variantes associadas às arritmias malignas, que permitem uma avaliação individualizada da indicação primária do CDI. Por muitos anos, a indicação do CDI como profilaxia primária na CMD levava em consideração a FE e classe funcional, sendo recomendado apenas para pacientes com FE<35%, sintomas e expectativa de vida maior que 1 ano. No entanto, estudos demonstram que pacientes com genótipos específicos se beneficiariam do CDI primário, mesmo na ausência de disfunção ventricular grave.^7^ A *Heart Rhythm Society* sugere a indicação do CDI para portadores de variantes patogênicas no gene *LMNA* (mutação *non-missense* ), na presença de dois ou mais dos seguintes fatores: FE <45% na primeira avaliação, taquicardia ventricular não sustentada (TVNS) e sexo masculino (grau de recomendação classe IIa – benefício é maior que o risco; mais estudos são necessários; tratamento é razoável).^13^

A identificação de variantes genéticas e sua influência no nível proteico possibilita a compreensão dos mecanismos fisiopatológicos específicos e abre oportunidades para o desenvolvimento de terapias direcionadas. Na CMD por *LMNA* , por exemplo, estudos em animais mostraram que há ativação aumentada da p38 MAP quinase. O uso de um inibidor da p38 MAP quinase (ARRY-371797) inibiu esse efeito e preveniu a dilatação ventricular.^34^ Essa droga atualmente está em avaliação em um estudo randomizado de fase 3 (NCT 03439514) para CMD por *LMNA.* ^7^

Além disso, novas técnicas de edição gênica, como CRISPR/CAS9 ( *clustered regularly interspaced short palindromic repeats* ), mostram-se alternativas terapêuticas promissoras.^7 , 35^

Em pacientes com variantes patogênicas em genes desmossomais foi comprovado o papel da atividade física no desenvolvimento e progressão da doença e na ocorrência arritmias malignas. Portanto, a recomendação atual é a abstenção de atividade física competitiva ou de alta intensidade nesses pacientes.^36^

### A realidade brasileira

Atualmente no Brasil, os testes não são disponíveis para todos, com maiores dificuldades de uso no Sistema Único de Saúde. As principais dificuldades enfrentadas para incorporação de tais recursos na prática clínica são a escassez de profissionais capacitados, falta de formação na área dentro dos programas de residência médica em cardiologia e a dificuldade de financiamento.^37^ Porém, cabe lembrar que a aplicação da abordagem de testagem genética e rastreamento familiar mostrou-se custo-efetiva e tem o potencial de tornar o sistema público de saúde muito mais proativo e não apenas reativo.^38^ Ressaltamos a experiência nacional com iniciativas patrocinadas pelo Ministério da Saúde brasileiro, no âmbito do SUS, que buscam uma maior compreensão das cardiopatias hereditárias, como a Rede Nacional de Genômica Cardiovascular (RENOMICA) e o Centro de Medicina de Precisão em Cardiologia (Cardiogen), financiados pelos projetos Genomas Brasil e Mapa Genoma Brasil.^39 - 42^

## Conclusão

A avaliação genética na CMD é fundamental por proporcionar informações prognósticas ao probando e oportunidades de diagnóstico e tratamento precoces em familiares, além de guiar a indicação de intervenções específicas. O adequado conhecimento da indicação e interpretação pelos médicos cardiologistas é fundamental para o uso efetivo desta técnica. A redução de custos e o consequente aumento da disponibilidade dos testes, proporcionou que a genética cardiovascular se faça cada vez mais presente na prática clínica. Perspectivas futuras incluem estudos que refinem a avaliação diagnóstica e prognóstica na CMD, bem como o desenvolvimento de terapias-alvo.
