# Supplementary material for: Desafios e Aplicações dos Testes Genéticos na Cardiomiopatia Dilatada: Genótipo, Fenótipo e Implicações Clínicas
Source: Arq Bras Cardiol. 2023 Nov 14;120(10):e20230174. [Article in Portuguese] doi: 10.36660/abc.20230174 (PMC10697682; doi:10.36660/abc.20230174)
Supplement: Translation

# Translation: Challenges and Applications of Genetic Testing in Dilated Cardiomyopathy: Genotype, Phenotype and Clinical Implications

**Authors:** Silas Ramos Furquim, Bianca Linnenkamp, Natália Quintella Sangiorgi Olivetti, Fernando Rabioglio Giugni, Layara Fernanda Vicente Pereira Lipari, Fernanda Almeida Andrade, José Eduardo Krieger

**Affiliations:** 1 Brazil Instituto do Coração do Hospital das Clínicas da Faculdade de Medicina da Universidade de São Paulo, São Paulo, SP – Brazil

**Keywords:** Cardiomyopathy, Dilated, Genetics, Genetic Testing

## Abstract

Genetic tests for dilated cardiomyopathy (DCM) have a diagnostic yield of up to 40%, but there is significant genetic heterogeneity and other challenges, such as variable expressivity and incomplete penetrance. Pedigree analysis is essential for distinguishing between sporadic and familial DCM cases by assessing family history. Familial DCM yields higher results in genetic testing, but sporadic DCM does not rule out the possibility of a genetic cause. Some genes have specific phenotypes, with the Lamin gene ( *LMNA* ) being associated with a phenotype of malignant arrhythmias and advanced heart failure (HF). The presence of a causal genetic variant can also aid in prognostic evaluation, identifying more severe cases with lower rates of reverse remodeling (RR) compared to individuals with a negative genotype. Current guidelines recommend genetic evaluation and counseling for individuals with DCM, along with cascade screening in first-degree relatives in cases where one or more variants are identified, offering an opportunity for early diagnosis and treatment. Relatives with a positive genotype and negative phenotype are candidates for serial evaluation, with frequency varying by age. Genotype also assists in individualized recommendations for implantable cardioverter-defibrillator (ICD) placement and advice regarding physical activity and family planning. Ongoing studies are progressively elucidating the details of genotype/phenotype relationships for a large number of variants, making molecular genetics increasingly integrated into clinical practice.

**Central Illustration:** : Challenges and Applications of Genetic Testing in Dilated Cardiomyopathy: Genotype, Phenotype and Clinical Implications

ACTC1:actin alpha cardiac muscle 1; BAG3: BAG cochaperone 3; DNA: Deoxyribonucleic Acid; DES: Desmin; DMD: Distrofin; DSC2: desmocollin 2; DSG2: desmoglein 2; DSP: desmoplakin; EMD: emerin; FLNC: Filamin C; LMNA: Lamin A/C; MYBPC3:myosin binding protein C3; MYH7: myosin heavy chain 7; PLN: phospholamban; RBM20: RNA-binding motif protein-20; SCN5A: sodium voltage-gated channel; alpha subunit 5; TMEM43:transmembrane protein 43; TNNI3:troponin I3; TNNT2: Troponin T; TPM1: Tropomiosin; TTN: Titin.

## Introduction

Dilated cardiomyopathy (DCM) is a condition characterized by the enlargement of the left ventricle (LV) and reduced ejection fraction (EF) in the absence of secondary causes, such as myocardial ischemia, arterial hypertension, primary valve disease, or congenital heart diseases.^1 - 4^DCM is relatively common, with a prevalence ranging from 1/250 to 1/500 in the general population,^4 , 5^and it is the leading cause of heart transplantation worldwide.^6^When the etiology of DCM is not well established, investigating a genetic cause becomes relevant. In up to 40% of DCM patients, a pathogenic or likely pathogenic genetic variant that could explain the cardiac phenotype is found. This highlights the importance of genetic testing in this population. However, interpreting genetic test results in DCM can be challenging, considering the wide variety of involved genes, incomplete genetic penetrance in the clinical phenotype, and significant heterogeneity in variant expression within the clinical presentation.^3 , 5^Most genes associated with DCM do not exclusively cause this phenotype and are related to other cardiac diseases, such as hypertrophic cardiomyopathy (HCM), arrhythmogenic cardiomyopathy, or channelopathies, leading to overlapping phenotypes.^5 , 7 , 8^Identifying a pathogenic variant related to DCM is crucial for supporting the diagnosis, refining prognostic assessment, and, in cases of positive tests in asymptomatic patients, enabling clinical screening, early diagnosis, and treatment. Determining the most appropriate genetic testing method to request, accurately interpreting the identified variants, and understanding the practical implications of the results are current challenges in Precision Medicine in Cardiology. This review will address these aspects in light of recent publications in the field.

### Definition of sporadic and familial DCM

Familial DCM is defined when two or more family members meet the criteria for DCM or when the index case with DCM has a first-degree relative who experienced sudden death younger than 35 years old or has confirmed DCM through autopsy.^4 , 9 , 10^When DCM is familial, the probability of finding a pathogenic genetic variant associated with the phenotype is higher, and therefore, the genetic testing yield is greater. However, even in cases of apparently sporadic DCM (without an evident family history), the possibility of a genetic cause is not ruled out, considering the potential for *de novo* mutations.^8 , 9^The 2022 guidelines from the American Heart Association (AHA) / American College of Cardiology (ACC) recommend evaluating the family history of at least three generations, ideally as a human pedigree.^3^Basic steps for constructing a pedigree can be found in Table 1 and Figure 1 .

**Table 1:** – Main information and rules required to construct the pedigree


Identify the name of the proband (patient under evaluation), date of birth, age of the proband during assessment, and date of evaluation.
Build the pedigree with information from at least three generations from the proband and include the proband's first-degree relatives (parents, siblings, and offspring) and second-degree relatives (grandparents, uncles and aunts, nephews and nieces and grandchildren).
Indicate the proband's place of origin and family ancestry, in addition to asking whether there is known consanguinity in the family. Consanguinity is not always known; however, this possibility should be suspected when the parents are from a city with few inhabitants.
Identify known diseases and other findings through captions.
Position older individuals to the left and younger individuals to the right.
Indicate the age and cause of death of deceased individuals.
Number generations using Roman numerals to facilitate the identification of individuals in the family.
Signal individuals who have undergone genetic testing and indicate the result.

**Figure 1:** – Universal symbols utilized to build a pedigree. Adapted from Kim et al. ^43^

### When to perform genetic testing

The recommendations for genetic testing can be found in the latest guidelines for heart failure from the American Heart Association (AHA), the American College of Cardiology (ACC), the European Society of Cardiology (ESC), and the Brazilian Society of Cardiology (SBC). These guidelines recommend genetic evaluation for patients with DCM, along with genetic counseling.^3 , 4 , 11^Genetic testing can also be useful in borderline cases, such as discriminating between DCM and peripartum cardiomyopathy or arrhythmogenic left ventricular cardiomyopathy. Genetic testing can also identify differential diagnoses that require specific treatments, such as cardiac amyloidosis, which, in later stages, can present with reduced EF.^12^Additionally, genetic testing is indicated in decisions about interventions, such as placing an implantable cardioverter-defibrillator (ICD) for primary prevention.^13^It is important to note that genetic testing should be performed on the most affected family member, with a well-defined phenotype, to increase the test yield and subsequently allow cascade family screening.^14^

### Which Genetic Test to order

There is a diversity of genes related to DCM, and a method that simultaneously and rapidly analyzes key involved genes is necessary for efficient evaluation. The emergence of a new sequencing technology called Next-Generation Sequencing (NGS) has enabled high-throughput parallel sequencing of multiple genes, making these tests more accessible and allowing their integration into the specialist’s routine.

The use of NGS panels for the analysis of pre-selected genes associated with a specific phenotype has revolutionized clinical practice by combining greater speed and efficiency, and it is the recommended method by expert consensus for genetic testing in cardiology.^15^The list of genes should be updated based on scientific knowledge, and it is essential that the chosen NGS panel for investigating DCM includes at least the most frequently associated genes, as presented in Table 2 .^15 , 16^

**Table 2:** – Main genes to be sequenced in DCM

Gene	Frequency	Gene	Frequency	Gene	Frequency
*TTN*	18-25%	*MYBPC3*	2%	*DES*	<1%
*DSG2*	4–15%	*FLNC*	0–3%	*TMEM43*	<1%
*DSP*	1-13%	*ACTC1*	<1%	*TAZ*	Unknown
*PLN*	0–12%	*LDB3*	<1%	*BAG3*	Unknown
*LMNA*	6%	*TNNC1*	<1%	*RBM20*	Unknown
*MYH6*	4%	*TNNI3*	<1%	*DSC2*	Unknown
*MYH7*	4%	*TNNT2*	<1%	*DMD*	Unknown
*SCN5A*	0–2%	*TPM1*	<1%	*EMD*	Unknown

Broader tests, such as whole-exome sequencing and whole-genome sequencing, also rely on NGS technology and analyze all known genes. However, they come with the disadvantages of higher cost and longer processing time.^5 , 16^

### Genetic Testing Yield in DCM

The yield of genetic testing for identifying a pathogenic or likely pathogenic variant related to DCM ranges from 15% to 40%,^3^depending on factors such as a positive family history, the presence of comorbidities, and electrocardiogram (ECG) features.^17^Escobar-Lopez et al. proposed a scoring system to estimate the likelihood of genetic testing positivity, known as the Madrid Score ( Table 3 ). This score assesses the presence of skeletal myopathy, family history of DCM, low voltage on the ECG, the absence of hypertension, and the absence of left bundle branch block on the ECG. The presence of four or more of these factors can lead to a genetic testing positivity rate of up to 79% ( Figure 2 ).^17^

**Table 3:** – Madrid’s score. Predictors of positive genetic test in DCM

Predictors of positive genetic test	
Skeletal myopathy	1 point
Family history of DCM	1 point
Low voltage ECG	1 point
Absence of hypertension	1 point
Absence of LBBB	1 point
**Score: 0 to 5 points**	

DCM: dilated cardiomyopathy; ECG: electrocardiogram; LBBB: left bundle branch block. Adapted from Escobar-Lopez L. ^17^

**Figure 2:** – Genetic test yield according to the Madrid score category. Adapted from Escobar-Lopez L. ^17^

### Genetic variants interpretation

It is essential to emphasize that the presence of a genetic variant should be evaluated with great caution. Finding a genetic variant in a cardiomyopathy gene, by itself, is not sufficient to assert causality regarding the clinical phenotype. When conducting a genetic test, even in a healthy population, the presence of genetic variants in genes related to cardiomyopathies is common. It should be considered that these variants may be benign. Therefore, a crucial point is determining the pathogenicity of the variant. To define the pathogenicity of a variant, it is necessary to assess the strength of the association between the gene and cardiomyopathy. This can be supported by a tool that curates this relationship, such as ClinGen (www.clingenome.org), and following the criteria proposed by the American College of Medical Genetics and Genomics (ACMG).^18^Determining variant pathogenicity is complex and includes various pieces of information, such as the variant’s frequency in population genetic databases like the Genome Aggregation Database (GnomAD) and the Brazilian Online Archive of Mutations (ABraOM), biochemical characteristics, *in silico* predictions, investigation of previous reports linking the variant to the disease, allelic data, and family segregation data. It is essential to consider the type of mutation (missense, insertion, deletion, nonsense) and the variant’s effect on the protein (shortening, premature termination, alteration in phosphorylation). Additionally, one should consider whether the described pathogenicity mechanism for that gene aligns with the variant’s effect on the protein.^18^

Variants can be classified into five categories: class 5 (pathogenic), class 4 (likely pathogenic), class 3 (variants of uncertain significance or VUS), class 2 (likely benign), or class 1 (benign).^18^Pathogenic variants are considered causal, while likely pathogenic variants have a 90% chance of being causal. Uncertain significance variants (VUS) represent an area of greater uncertainty regarding pathogenicity, which can range from 10% to 90% likelihood of being causal ( Table 4 ).^18^Some strategies for reclassifying a VUS include conducting functional studies that test the variant’s effect on the protein, assessing family segregation of the variant in first-degree relatives, and conducting periodic literature searches.^18^It is important to note that variant classification is a dynamic process that depends on up-to-date knowledge about the specific variant. Therefore, reclassifications are ongoing, and searching for new information is essential.

**Table 4:** – Variant classification according to ACMG criteria

Classification of pathogenicity	class	probability
Benign	class 1	< 5%
Likely Benign	class 2	< 10%
Variant of uncertain significance (VUS)	class 3	10 - 90%
Likely Pathogenic	class 4	> 90%
Pathogenic	class 5	> 95%

ACMG: American College of Medical Genetics and Genomics. Source Richards et al. ^18^

### Different Genes Involved in DCM

Unlike HCM, where 70% of variants are found in *MYH7* and *MYBPC3* genes,^19^DCM exhibits greater heterogeneity, with over 50 genes with described association with the phenotype.^7^These genes encode proteins that function in various structures of cardiomyocytes ( Table 5 ). For example, Lamin A/C ( *LMNA* ) and RNA-binding motif protein-20 ( *RBM20* ) are found in the cell nucleus and are present in 8% of DCM patients.^10 , 20^In the sarcoplasmic reticulum, phospholamban ( *PLN* ) is found, and when unphosphorylated, it inhibits the sarcoplasmic reticulum Ca2+-ATPase ( *SERCA* ).^10 , 21^In the cytoskeleton, variants in the Filamin C ( *FLNC* ), desmin ( *DES* ), and dystrophin ( *DMD* ) genes account for approximately 11% of cases.^20^In the sarcomere, there are genes such as *TTN, MYH7, TNNT2, TPM1,* and *MYBPC3.*

**Table 5:** – Description of genes related to DCM, encoded proteins, and the region of the cardiomyocyte where they are found

Gene	Protein	Region
LMNA	Lamin A/C	Nuclear envelope
RBM20	RNA-binding motif protein-20	Nucleus
PLN	Phospholamban	Sarcoplasmic reticulum
FLNC	Filamin C	Cytoskeleton
DES	Desmin	Cytoskeleton
DMD	Dystrohin	Cytoskeleton
TTN	Titin	Sarcomere
MYH7	Beta-cardiac/slow skeletal myosin heavy chain	Sarcomere
TNNT2	Troponin T2	Sarcomere
TPM1	Tropomyosin 1	Sarcomere
MYBPC3	Myosin-binding protein C 3	Sarcomere
SCN5A	Sodium channel, voltage-gated, type V, alpha subunit	Cell membrane

The *TTN* gene, which encodes the titin protein, is the most commonly implicated in DCM, with truncating variants accounting for 25% of familial and 18% of sporadic DCM cases.^22^Variants in the *MYH7, TNNT2,* and *TPM1* genes have a prevalence of 5 to 10%,^23^and although *MYBPC3* is more specific to HCM, it is also found in DCM.^24^Additionally, the main sodium channel of the heart, encoded by the *SCN5A* gene, is located in the cell membrane. While variants in this gene are well-described in arrhythmias such as long QT syndrome type 3, and Brugada syndrome, missense variants are also associated with DCM phenotype.^10^The wide array of implicated genes, coupled with significant phenotypic overlap, makes the genetic evaluation of DCM challenging.^20^

### Clinical manifestations in specific variants

The clinical manifestations of specific variants in DCM are highly heterogeneous, meaning the same gene can be responsible for different phenotypes. For instance, variants in the *TNNT2* gene can manifest as hypertrophic, dilated, or restrictive cardiomyopathy. This clinical variation can be explained by epigenetics, which involve interactions between the specific variant, an individual’s genetics, and external factors such as hypertension, alcoholism, lifestyle, physical exercise, tachycardia, chemotherapy, or inflammation.^10 , 20^

Some genes, like *PLN, FLNC,* and *LMNA* , are more directly associated with aggressive phenotypes, such as a higher risk for ventricular arrhythmias, sudden death, and a worse prognosis. The *LMNA* gene is particularly linked to a specific phenotype, currently referred to as laminopathy, characterized by early-onset left ventricular dysfunction (around 30 to 40 years of age), early-onset conduction disorders (like complete atrioventricular block), atrial fibrillation in young individuals, complex ventricular arrhythmias, and high risk of sudden cardiac death, even in the absence of left ventricular dysfunction.^9 , 20 , 25 , 26^As a result of this association, there are specific recommendations for competitive sports restriction and primary prophylaxis with an ICD in this population.^13^

Additionally, pathogenic and likely pathogenic variants in desmosomal genes, such as desmoplakin ( *DSP* ), were initially linked to arrhythmogenic right ventricular cardiomyopathy, but new evidence also demonstrates an association with the DCM phenotype.^27^Along with the *FLNC* and *LMNA* genes, they are the main causes of arrhythmogenic cardiomyopathy in the predominantly left ventricular form, characterized by life-threatening arrhythmias that occur at an earlier stage, disproportionate to the degree of left ventricular dysfunction.^20^

The *TTN* gene encodes the titin protein, which is important for the passive elasticity of myocardial tissue. Loss-of-function variants in this gene play a well-established role in DCM pathogenesis, while missense variants are common and mostly considered benign.^20^

Finally, the *DMD* gene, related to muscular dystrophies, is associated with a typical clinical manifestation of progressive muscle weakness. The incidence of cardiomyopathy increases with age, especially in males, being over 90% by the age of 18. The ECG shows a classic pattern with tall R waves and increased R/S amplitude in V1, Q waves in left precordial leads, right-axis deviation, or complete right bundle branch block.^28^

### Prognostic impact of genetic testing

Patients with DCM and pathogenic or likely pathogenic variants have worse clinical outcomes, especially regarding the risk of malignant arrhythmias and advanced heart failure, compared to DCM patients with a negative genotype. The risk is particularly higher in those with EF ≤ 35%.^29^However, there are variations among the affected genes; *PLN, LMNA* , and *FLNC* carry a higher risk of malignant arrhythmias, even with EF > 35%.^29^Patients with laminopathies have a mortality rate of approximately 12% in 4 years and a higher need for heart transplantation by the age of 45.^9 , 20 , 25^Variants in *FLNC* are associated with a high risk of malignant ventricular arrhythmias and sudden death, with rates of 15-20% for ventricular arrhythmias or sudden death in 5 years follow-up and 6% mortality rate.^30^ *PLN* variants are linked to more severe forms of cardiomyopathy, with malignant arrhythmias, rapid progression to advanced heart failure, and need for heart transplantation.^20^Among the mentioned genes, variants in *TTN* show better prognosis, with lower rates of malignant arrhythmias and higher incidence of RR.^29^

### Reverse remodeling

DCM is a dynamic disease that can show an improvement in EF in response to treatment, a process known as RR. This improvement occurs in approximately 40% of cases.^31^Studies have investigated the relationship between the genetic basis of DCM and RR, demonstrating that patients with a positive genotype have lower RR rates, especially those carrying desmosomal variants ( *PKP2, DSG2, DSC2, JUP, DSP* ), variants related to the nuclear envelope ( *LMNA* ), and variants in sarcomeric genes ( *MYH7, MYBPC3* ).^29^

Therefore, knowing patients’ genotypes can provide important information on risk stratification and prognostic prediction in DCM, leading to more frequent monitoring, optimization of therapy, and earlier evaluation for mechanical ventricular assist devices or heart transplantation if there is an unfavorable clinical progression of heart failure.

### Family screening and follow-up

Once a pathogenic or likely pathogenic variant is identified in the index case, cascade screening of family members should be performed, as recommended by the major heart failure guidelines.^3 , 4 , 11^This assessment aims to identify relatives at risk of developing the condition carrying the variant and allows for detecting individuals with early asymptomatic disease. In a study that identified pathogenic or likely pathogenic variants in heart transplant patients with DCM, family screening described pathogenic variants in 39.6% of the relatives, and most of these (52.6%) did not show clinical signs of the disease.^32^Diagnosing relatives is useful for initiating early treatment and preventing disease progression and sudden death.^3 , 4 , 14^Key data are summarized in the central figure.

Genetic testing is recommended in first-degree relatives aged 10 to 12 years when a specific variant is identified in the index case.^20 , 33^In this case, Sanger sequencing can be used instead of NGS, targeting only the variant of interest, saving time and resources.^20^

Age is an important factor in the development of cardiomyopathies;^14^in a cohort evaluated with truncating variants in *TTN* , for example, penetrance at the age of 40 was over 95%.^22^Therefore, identifying a genotype-positive relative with a negative phenotype requires serial evaluation, especially while they are at ages with greatest risk for developing the phenotype.^3 , 14 , 20^

In addition to genetic testing, relatives also need a clinical consultation with genetic counseling, medical history, and physical examination, along with complementary tests such as echocardiography, ECG, and, if necessary, Holter monitoring and stress testing.^10^The result of genetic testing can even determine the frequency of clinical follow-up and the need for repeating these tests.^4^The frequency of evaluation of family members with a positive genotype and a negative phenotype varies depending on age and the heart disease of interest. The Heart Failure Society of America suggests different intervals for various age groups, as outlined in Table 6 .^14^

**Table 6:** – Periodicity of clinical evaluation in a first-degree relative of an index case, with a genetic variant related to DCM identified

Age	0-5 years	6-12 years	13-19 years	20-50 years	>50 years
Periodicity of clinical evaluation	Yearly	1-2 years	1-3 years	2-3 years	5 years

There are no recommendations to start pharmacological treatment in patients with genetic variants and negative phenotype; an exception is the presence of a variant in *LMNA* , which indicates ICD placement even in asymptomatic phases, in the presence of other criteria discussed ahead.

On the other hand, relatives with a negative genotype have a low probability of developing the disease and do not require serial assessment, minimizing the burden of potential future cardiac involvement.^14^

When a pathogenic or likely pathogenic variant is not found in the index case, first-degree relatives should not undergo genetic testing and should be referred for serial clinical evaluation while they are at risk of developing the phenotype, according to their age.^33^

Challenging situations persist, such as incomplete penetrance, expression of different phenotypes among family members, and uncertainty about when the disease will manifest.

### Targeted therapies and specific recommendations

The presence of specific genetic variants can entail particular risks, leading to distinct treatment and management recommendations. This is the case for variants associated with malignant arrhythmias, which allow for an individualized assessment of primary ICD indication. For many years, the indication for primary ICD in DCM considered the EF and the functional class, being recommended only for patients with EF <35%, symptomatic, and with life expectancy greater than 1 year. However, studies demonstrate that patients with specific genotypes would benefit from primary ICD, even without severe ventricular dysfunction.^7^The Heart Rhythm Society suggests ICD placement for carriers of pathogenic variants in the *LMNA* gene (non-missense mutation) in the presence of two or more of the following factors: EF <45% on initial evaluation, nonsustained ventricular tachycardia (NSVT), and male sex (Class IIa recommendation – the benefit is greater than risk; more studies are needed; treatment is reasonable).^13^

Identifying genetic variants and their influence on the protein level allows for understanding specific pathophysiological mechanisms and opens up opportunities for developing targeted therapies. In *LMNA* -related DCM, for example, animal studies have shown increased activation of p38 MAP kinase. Using a p38 MAP kinase inhibitor (ARRY-371797) inhibited this effect and prevented ventricular dilation.^34^This drug is currently being evaluated in a phase 3 randomized study (NCT 03439514) for *LMNA* -related DCM.^7^

Furthermore, new gene-editing techniques such as CRISPR/CAS9 (clustered regularly interspaced short palindromic repeats) are promising therapeutic alternatives.^7 , 35^

In patients with pathogenic variants in desmosomal genes, the role of physical activity in the development and progression of the disease and in the occurrence of malignant arrhythmias has been proven. Therefore, the current recommendation is to abstain from competitive or high-intensity physical activity in these patients.^36^

### The brazilian reality

Currently, genetic tests are not widely available in Brazil, with greater difficulties in accessing them through the public health system (Sistema Único de Saúde - SUS). The main challenges faced in incorporating such resources into clinical practice are the shortage of qualified professionals, lack of training in the field within cardiology residency programs, and funding difficulties.^37^However, it is worth noting that the application of genetic testing and family screening approaches has shown cost-effectiveness and has the potential to make the public health system more proactive rather than reactive.^38^We highlight the national experience with initiatives sponsored by the Brazilian Ministry of Health, under the SUS, aimed at a better understanding of hereditary heart diseases, such as the National Cardiovascular Genomics Network (Rede Nacional de Genômica Cardiovascular or RENOMICA) and the Center for Precision Medicine in Cardiology (Cardiogen), funded by the *Genomas Brasil* and *Mapa Genoma Brasil* projects.^39 - 42^

## Conclusion

Genetic assessment in DCM is crucial for providing prognostic information to the proband and opportunities for early diagnosis and treatment in family members, as well as guiding specific interventions. Adequate knowledge of indications and interpretation by cardiologists is essential for the effective use of this technique. The reduction in costs and the consequent increase in test availability have made cardiovascular genetics increasingly present in clinical practice. Future prospects include studies that refine diagnostic and prognostic evaluation in DCM, as well as the development of targeted therapies.
